# Book Reviews

**Published:** 1824-04

**Authors:** 


### CRITICAL ANALYSIS

OF

### ENGLISH AND FOREIGN LITERATURE

RELATIVE TO THE VARIOUS BRANCHES OF

### Medical Science.

Quæ laudanda forent, et quæ culpanda, vicissim

Illa, prius, cretâ; mox hæc, carbone, *notamus*.—PERSIUS.

### DIVISION I.

### ENGLISH.

ART. I.—*Essay on the Effects of Iodine on the Human Constitution: with Practical Observations on its Use in the Cure of Bronchocele, Scrophula, and the Tuberculous Diseases of the Chest and Abdomen.* By W. GAIRDNER, M.D.—8vo. pp. 64. London: T. and G Underwood. 1824.

WE lately took an opportunity to direct the attention of our readers to the principal facts, which had then been made public, relative to the medicinal agencies of iodine. We had occasion to regret the imperfect nature of our former account, and therefore embrace with satisfaction the means presented to us, by the work at the head of this article, of rendering our history of this powerful drug somewhat more complete. We take the liberty to state, in commencing, that we have (in conformity with a general custom of ours) *read* the book we purpose to review,—and read it, too, with the gratification which always accompanies the attainment of an object. We have heard of iodine being given, —nay, we have even prescribed it ourselves; but, in the former case, the accounts were vague and contradictory, and, in the latter, the practice was timid: and, perhaps on that account, unavailing; for we confess that we were able to gather, from the histories of cases published, neither what indications the medicine was calculated to fulfil,—what good was to be hoped from it, nor what evil to be dreaded. Such was the uncomfortable state in which we sat down to peruse Dr. GAIRDNER's essay; and we rose from it with the conviction that he had brought together, in a concise and well-digested form, all we were anxious to be informed of, and which we had looked for, in vain, in the various continental publications on this subject. Probably some of our readers may be similarly circumstanced; and, if so, we can refer them with confidence to the work before us; or, failing that, we solicit their attention to the following analysis, in which it may be anticipated, from what is above mentioned, that we shall deal largely in quotation, and but sparingly in comment.

Certain maritime productions, as the *fucus vesiculosus* and sponge, have long enjoyed some repute in the cure of goitre, although they have been regarded, by the majority of practitioners, as extremely uncertain, if not altogether inert. We suspect that this unfavourable opinion of the remedy, as generally obtained, is but too well founded; and the following remarks, introduced by the author as a note, will perhaps serve to explain the causes of its failure:

"The total inefficacy of this medicine in the hands of British practitioners, while its virtues are so palpable and evident at Geneva, that not only physicians, but also the inhabitants in general, are convinced of their reality, had always surprised me. I was at a loss to account for testimony so contradictory. It seemed as if medicine were a science so uncertain and futile, that its plainest facts depend more on the authority of name than on the substantial evidence of observation and experiment. I lately obtained an explanation of this difficulty, from a quarter in which I can place implicit reliance. It seems that the chemists are much in the habit of substituting charcoal for burnt sponge; of which, an undeniable proof is the fact that burnt sponge is sold at an inferior rate to the same article before it has undergone the process of combustion.—I may also be allowed to state in this place, that I have sent prescriptions for the hydriodate of potass to several chemists in London; that my prescriptions were said to have been made up; but that, a few days afterwards, when I called at their shops in order to examine the medicine, I discovered that they were not even aware of the existence of such a drug. If such frauds continue to be committed with impunity, the sick had better submit patiently to their pains, than have recourse to physicians, whose science is rendered unavailing for the profit of tradesmen." (p. 2, 3.)

It appears, however, that Dr. Coindet had been led, not merely to form a favourable opinion of the powers of the fucus and sponge in the treatment of goitre, but likewise to suspect that iodine might be the active ingredient common to both.

The successful result of his experiments gave rise to a certain degree of enthusiasm in a country where goitre constitutes so prevalent a deformity. This is the most unfortunate circumstance that can possibly be connected with the reputation of a new and powerful drug, and, as might naturally be expected, quickly led to its abuse, and to much mischief consequent thereupon. Many lost their lives, and many more suffered in health from its use, until at length the government, in order to check the growing evil, prohibited its sale, unless sanctioned by the signature of a regular physician.

It is justly observed by Dr. Gairdner, that nothing can tend more to assist us in forming an accurate estimate of its virtues, than a careful observation of the bad effects which flowed from its abuse. Some of the most remarkable of these we shall now proceed to give, in the words of the author.

" Some time after the introduction of iodine into practice, a few cases of severe spasmodic affection of the stomach and bowels occurred. They were attended with violent and incessant vomiting, excruciating pain of stomach and bowels, strong spasms of the back and legs. The tongue was commonly furred, and the bowels sometimes violently purged, at other times obstinately constipated. The pulse was generally extremely frequent, small, and depressed; the eyes sunk and hollow; the countenance ghastly and pale. These accidents were usually imputed by the patients to the iodine they had taken. The physicians, by whose advice the medicine had been given, would not allow this origin of the disease, till a repetition of similar cases determined that the sufferers were right. The vomiting, pain of the bowels, and the cramps of the legs, are extremely severe. They are also with the greatest difficulty allayed, continuing sometimes for many days, and renewed during weeks, and even months, after taking food. The legs sometimes swell in the first instance, and afterwards become rapidly thin and meagre. There is another symptom, which, though common to almost all diseases, is peculiarly the sign of this. The emaciation which attends this irregular action of iodine is so rapid and so extreme, as to strike terror into the minds both of patients and physicians. A magistrate of Geneva, high in office, robust, corpulent, and of an athletic form, was so much reduced in flesh, that he was not known by his oldest acquaintances. I have seen emaciation, in one case, proceed to such an extent in a short time as is almost incredible. A young English lady, at a boarding-school at Paris, had for some time been afflicted with goitre. Her brother was prosecuting the study of medicine there. With the characteristic zeal of a young man, as soon as he heard of the wonderful effects of iodine, he determined on making trial of its powers on his sister. He did not find much difficulty in persuading her to become the subject of his experiments; nor did he encounter more difficulty on the part of the French gouvernante, to whose care she was confided. The remedy succeeded, as usual, in greatly diminishing the tumor, and for some time no bad effects were apparent. A small hard knot only remained in the situation which had been occupied by a considerable swelling before; and the desire to get rid of this little tumor was the cause of the remedy having been pushed too far. Its deleterious effects first showed themselves by gnawing pain at the upper part of the stomach, great anxiety, and oppression. These symptoms were disregarded, and the remedy was persevered in for a week longer, during which time the patient became very much emaciated; she was frequently affected with vomiting, the pain of the abdomen became more frequent and more severe, and the thirst was very distressing. I was sent for early in the morning, in consequence of an alarming diarrhœa, which had come on during the night; and I found her in a deplorable condition, indeed. Her brother, and the mistress of the boarding-school, were so alarmed at the consequences of their conduct, that they were quite unfit to give any advice about her treatment; they could hardly, indeed, give me a coherent account of what had passed; and the poor young lady was therefore entrusted to the care of servants. She was then suffering the most excruciating pain at the stomach, violent cramps, and convulsive action of the muscles of the arms, back, and legs, from which she had scarcely any intermission. The vomiting and purging were almost incessant. The dejections were bloody, slimy, and very scanty, but at first had been copious and feculent. The matter vomited was of a dark-green colour, streaked with blood. The tongue was loaded with a thick crust, resembling in colour the matter vomited. The countenance was pale, contracted, and with that peculiar expression which announces abdominal suffering. The pulse was small, hard, and frequent,—scarcely, indeed, to be numbered. The whole appearance of the patient was such as to excite well-grounded fears for her life. Being quite unable to swallow, four grains of opium were directed to be thrown into the rectum; they were not, however, long retained, and were not productive of benefit. An anodyne embrocation was therefore applied to the pit of the stomach; fomentations to the feet; and, as soon as it could be got ready, she was placed in a warm bath. This so much quieted the irritation of the stomach, that she was enabled to swallow about thirty drops of laudanum, from which there was a decided alleviation of her sufferings for nearly an hour. During ten days she remained in a very doubtful state, subject to frequent severe attacks of diarrhœa, with intense pain of the bowels. Her emaciation during this time was most extraordinary. The expression of her French nurse, '*décharnée*,' was literally applicable to her. Her arms and body were almost fleshless; her breasts, which had been large, were now perfectly flat; the calves of her legs had quite disappeared; and her thighs were not much thicker than her wrists, when in health. I never witnessed any thing like such an extenuation in so short a space of time. By the steady and very liberal use of opium, she recovered to a certain degree; but, when I last saw her, many months after her illness, she remained subject to frequent violent spasms of the stomach, during which opium alone gave her relief. Her nervous system had been much shattered. She repeatedly declared to me that she seldom enjoyed an hour's respite from the most wretched depression of spirits, and, since her illness, had never felt any thing like her former buoyancy of mind. The few moments of ease she knew were purchased by large doses of laudanum, to the habitual use of which her sufferings had forced her. She was still very pale, and her emaciation, though much less, was yet very great. She was, indeed, a miserable monument of the effect of iodine. I heard of this young lady a few weeks ago: she was then much better, had in a great degree recovered her looks, and was able to leave off the use of opium almost entirely. Her stomach, however, still remained very weak, and obliged her to be very careful of her diet. The bronchocele had not returned; but the small hard swelling mentioned above remained still very sensible to the touch, but not evident to the eye." (p. 5—11.)

Such are the details of this interesting and instructive case in which the injurious effects of iodine, when injudiciously administered, are powerfully described. The anxiety and depression here described are, it appears, very frequent attendants on the improper use of iodine, and are regarded by our author as the peculiar effect of the medicine, and not merely the result of the concomitant debility; sometimes occurring in a lesser degree, even when the iodine operates in a salutary manner.

The nervous and muscular systems are next considered in their relations of the effects of iodine; and we are informed that, in some persons, it cannot be exhibited without giving rise to some of the "countless forms of inward nervous derangement." Among these, a certain degree of tremor is so characteristic, as to be regarded by the author as a measure of the constitutional influence of the remedy. This is described by Dr. Gairdner as generally beginning with a slight trembling of the hands, similar to that resulting from the poison of lead; afterwards the arms, legs, and back, become affected; while, in addition to these symptoms, there is derangement of the stomach and constipation of the bowels. With a view of detecting this affection in its incipient stage, the author recommends the practice of frequently obliging the patient to raise an empty glass, or other light body, to the mouth; by which means the smallest unsteadiness is rendered obvious. This affection obviously bears a strong resemblance to chorea; notwithstanding which, Dr. Gairdner regards them as differing in some essential points.

The external application of iodine has sometimes been accompanied with slight pain at the stomach, and copious bilious evacuations; but these never proceed to the degree of violence which marks its internal exhibition.

When the symptoms of cholera, detailed in the case we have quoted, are brought on by the employment of iodine, opium, in its different forms, is to be regarded, not only as the best, but almost as the *unicum remedium.* In the case mentioned by the author, a fourth of a grain of the acetate of morphia, was given every half-hour with great advantage; the other preparations of opium having entirely failed. In other instances, however, this has not answered in so satisfactory a manner. To the opium may be added bitters and astringents, but not until the disease has begun to abate in violence. Purgatives are represented as invariably and conspicuously injurious, however gentle. The warm bath is never to be neglected.

For the chronic stage of the disease, the administration of aperients and opium is recommended; and, for the spasms and nervous derangement, light diet, gentle exercise in the open air, and the warm bath. The use of wine and spirits, as well as the exhibition of the ethers and tinctures, are very strongly condemned.

Such are some of the evils arising from the injudicious use of this medicine, and the list is, no doubt, sufficiently extensive and alarming. Nevertheless, it appears as possible to control its energies, and disarm it of its dangerous properties, as to regulate the effects of any other equally powerful drug. Dr. Gairdner informs us that he has used it in "a great number" of cases, without ever meeting with any accident; having discontinued the medicine when the pulse became frequent, small, and depressed, with watchfulness, and flying pains of the joints, &c. Dr. Coindet has given it to 150 patients; Dr. Decarro, of Vienna, to 120; Dr. Erlingen, of Zurich, to seventy; Dr. Formey and Hufeland have prescribed it "extensively;" and all with beneficial results. It has also been used by many other practitioners, both in this country and on the continent; the result of whose practice is but partially known.

It is upon the absorbent system that the operation of iodine is most conspicuous, and in no disease is this more remarkable than in bronchocele. Our author proceeds:

"Decarro states, that one of his patients, thirty-eight years of age, after taking the remedy for seventeen days, had the circumference of his neck reduced from one foot seven inches and a half, to one foot three inches and three quarters. Dr. Coindet relates a case of a man, fifty years of age, in which this medicine, taken internally, reduced a very large goitre considerably in size, after six days' treatment only. An old woman, aged sixty-five, who took this medicine under my care for a goitre, with which she had been affected nearly forty years, had the circumference of her neck reduced from twenty-two inches to eighteen, on the twenty-fifth day. Such rapid diminution in the size of the tumor is not to be always expected. In some cases a whole month, and even more, elapses before any effect is visible. In general, however, the powers of the medicine are manifest at the end of the second week, and considerable progress towards cure has been made at the end of a month. I have endeavoured to find out whether there was any thing in the constitution of the different persons under my own observation, or in their state of health, which rendered them more or less apt to be affected by this medicine. I have not been very successful in this inquiry. But I found that, in two cases of women afflicted with extensive and very painful varix of the veins of all the extremities, the effect of iodine was produced with great difficulty. This fact seemed to coincide with the result of Mr. Magendie's very interesting experiments on absorption, and I accordingly desired one of the persons, to whom I have just alluded, to lose a little blood from the arm. The effect of the medicine was very much accelerated by this treatment; but a consequence I did not look for was also the result of it,—viz. the total and sudden disappearance of the varix, which had commenced during uterine gestation twelve years before. The goitre succeeded the varix after her delivery. I merely mention the facts of this case, which may suggest useful hints to those who may meet with a case similarly circumstanced. Since its occurrence, whenever the medicine is slow in its operation, provided the vessels be full and plethoric, I desire a little blood to be taken away from the arm, and I almost invariably find the action of the medicine much quickened. I have sometimes, also, thought that the cases in which blood was taken away were cured more easily, and with less suffering, than the others." (p. 35—38.)

The medicine, when administered "with propriety and caution," rarely produces any considerable effect upon the pulse, although it has the remarkable property of stimulating very powerfully the vessels of the tumor. In this remark Dr. Gairdner is supported by the testimony of other writers. Occasionally this symptom of local excitement proceeds so far as to require the abstraction of blood; and for this purpose topical bleeding is generally sufficient. In treating a case of bronchocele with iodine, this peculiarity of its operation is, of course, to be kept in mind; because, as is justly observed by the author, the occurrence of inflammation on an extensive surface in contact with the trachea, is by no means free from danger.

With respect to the forms and methods of administering this powerful medicine;—the hydriodate of potass, as recommended by Dr. Coindet, seems to have obtained a general preference. Dr. Gairdner informs us that the hydriodate of soda answers equally well, but states no reason why it should be preferred to the one in more general use. He has likewise given a trial of the iodates, but found them "at once more inert and more unmanageable."

"I have generally ordered half a dram of the hydriodate of potass to be united to an ounce and a half of axunge, and desired the patient to rub in a dram of this ointment over the surface of the tumor, night and morning. When the tumor is painful, it is not necessary to rub in. The ointment may be used in the manner recommended by Scattigna. All that is necessary is, to choose a portion of the surface of the body where the skin is very tender and thin, and simply to apply the ointment over night. For this purpose, almost any part of the body which is habitually covered may be chosen; but in the axilla, and in the inner surface of the thighs close to the scrotum, the absorption will be found most rapid." (p. 42.)

For internal exhibition, Dr. Gairdner gives a decided preference to the solution over the tincture: it is made by dissolving half a drachm of the hydriodate of potass in an ounce of distilled water, of which ten drops may be given for a dose, and the quantity gradually augmented to twenty or twenty-five; but this appears, from the evidence of our author, to be seldom requisite.

The decided effect produced by iodine on the absorbent system, as evinced in the cure of bronchocele, led to its employment in scrofula. The result of our author's observations is thus expressed:

"The first case of scrophula, in which I made use of this medicine, was that of a young lady, eighteen years of age, who had been affected by glandular swellings of the neck for nearly eight years. She used the solution of hydriodate of potass for a month; the dose varied from ten to twenty drops three times a-day, with occasional intermission of a day, when the absorption was going on rapidly. At the end of this time she had got perfectly rid of her swellings, and she now (two years since she took the medicine,) remains perfectly well. When she discontinued her drops, so far from having been incommoded by them, her health was certainly much improved. There remained several little fistulous sores, which required the assistance of the knife to heal them. The iodine is not equally efficacious in all cases of this kind. Great numbers, however, yield rapidly under its use; but many of them, also, resist its operation. I have never been able to assign even a plausible reason for this difference of its action in scrophula. In general, I have found such cases yield more readily to the internal than to the external use of iodine. The scrophulous glands of children are not so easily affected by iodine, as those of persons who have attained the age of puberty; and they are also more liable to a relapse." (p. 47, 48.)

Dr. Gairdner likewise mentions the case of a boy, of five years old, in whom "it was impossible to doubt, from the appearance of the child, that the mesenteric glands were enlarged." He had been weakly from the period of his birth, and had been gradually losing flesh and strength for two years, with enlargement of the abdomen and irregularity of the bowels. The author gave him twelve drops of the solution of the hydriodate of potass in the day, and gradually increased the quantity to twenty drops. At the end of five weeks he was restored to "comparative health," no other medicine having been given, except occasionally a few grains of rhubarb. In two other cases of a similar nature, considerable relief was afforded; but the result was not, upon the whole, so satisfactory as in the preceding instance.

Dr. Gairdner has used iodine in cases where he had reason to believe tubercles existed in the lungs; and he is inclined to believe it will be found of service in incipient stages of pulmonary consumption, but apprehends injury from its operation in more advanced stages of the disease.

The author has given, in an Appendix, a short notice of some of the best forms of administering iodine; and with these we shall conclude our extracts from his interesting little volume.

"I have here thrown into an Appendix a brief account of the different preparations of which I have had occasion to make mention. It is chiefly extracted from Magendie's Formulary, which will be found to contain sufficient directions for the chemical and pharmaceutical operations undergone by iodine.

*Tincture of Iodine.*

Take of Alcohol, of sp. gr. of .842, 1 oz.
Iodine, 39 gr. Dissolve.

"This preparation should not be long kept, as it readily undergoes alteration and decomposition. Alcohol varies in its solvent power of iodine, according to its degree of concentration. The frequent opening of the vessels, therefore, in which it is kept, must occasion a change in the quality of the tincture, by allowing the evaporation of the spirit, and thus occasioning a diffusion of undissolved iodine through this preparation. Mr. Magendie seems also to fear that a decomposition of the alcohol may take place, from the superior affinity of iodine for hydrogen. Altogether this is certainly the most objectionable form in which iodine is used.

*Solution of Hydriodate of Potass.*

Take of distilled Water, 1 oz.
Hydriodate of Potass, 30 gr. Dissolve.

"I have generally prescribed these two preparations in cinnamon or mint water, in which form they are seldom disagreeable to the stomach. I have avoided, as much as possible, joining them to any tinctures or infusions, as we are yet in a great degree unacquainted with the chemical habits of iodine and the different vegetable substances. It will be sometimes, however, found advisable to use tonics with iodine.

*Ointment of Hydriodate of Potass.*

Take of Hydriodate of Potass, ½ dr.

Axunge, 1½ oz. Mix."*

---

### DIVISION II.

### FOREIGN.

ART. II.—*An Essay concerning Tussis Convulsiva, or Whooping-Cough; with Observations on the Diseases of Children.* By BENJAMIN WATERHOUSE, M.D. Member of many Medical, Philosophical, and Literary Societies in Europe and America; and late Professor of the Theory and Practice of Physic in the University of Cambridge.—pp. 152. Boston (America), 1822.

[Concluded from page 253.]

DR. WATERHOUSE, though he delights in the term *whoop*, is yet strangely puzzled with the word *cough*, and he would evidently have been better pleased to call the affection the *whooping disease.* "We never could admit," he says, "that the convulsion so distressing in this distemper was a true cough, but rather

* While this sheet was passing the press, we received a foreign Journal, containing some remarks on Iodine, which will be given in our "INTELLIGENCE."

a nervous affection, operating on parts previously disposed to spasms." "Writers," he adds, "have used the term *cough*, not being able, probably, to find a more correct term. There is no fact in physiology or pathology of which I am more certain, than that the clangorous whoop peculiar to the *tussis convulsiva* is not owing to the sudden rush of air into the exhausted lungs. On sudden waking from profound sleep, the lungs have neither time nor opportunity of producing the vacuum spoken of by some writers; their structure would not sustain such violence." He then goes on to state, (page 56,) "that, as he believes whooping-cough has its primary seat in the œsophagus, stomach, and diaphragm, according to the stages of it, and as *hiccup* takes its rise from the same parts, it may be profitable to extend the inquiry to the nature and seat of that symptom." Upon this subject, however, the author does not throw any new light. He inclines to the belief that hiccup is a spasmodic concussion of the diaphragm, that "*nobilissimus, post cor, musculus;*" and he is satisfied "that the peculiar clang, or sonorous whoop, in tussis convulsiva, partakes fully as much of the nature of a *diaphragmatic hiccup* as of a *pulmonic cough.*"

In the following paragraph, the author sums up his views of the nature of whooping-cough; and by these the reader will be apprised that it is taken out of the class of *thoracic*, and placed by Dr. Waterhouse in that of *abdominal* affections. The fortune of hooping-cough in this respect appears to be very singular: our readers may remember that, a short time ago, (vol. xlviii. page 478,) we laid before them the opinion of one of our correspondents, (Dr. WEBSTER,) that "the actual seat of this complaint may be in the head, and that the affection of the respiratory organs is only to be considered as secondary." Dr. Waterhouse's notions, however, are very different.

"Our own observation and personal feelings will not allow us to liken this disorder to any idiopathic affection of the lungs. It appears to me to be a spasmodico-convulsive action, arising from causes extra pulmonic. It seems to occupy, principally, the œsophagus and diaphragm; and, in very violent and long continued fits, spells, or paroxysms of coughing, it occupies that upper portion of the stomach, of which we have so often spoken; and, at last, the lower and more capacious part of it, when a vomiting ensues. In this action, the stomach throws up a great quantity of phlegm, resembling pure lymph, and now and then some remains of food, and then the commotion subsides: the lungs inspire and expire as before, and the countenance resumes its healthy aspect." (p. 59.)

The fourth chapter is dedicated to some general researches concerning *epidemic diseases* and their causes, and more particularly concerning the causes of whooping-cough.

"We have not been able," says our author, "to trace the whooping-cough to any local cause; nor to any known peculiarity in the seasons. It appears in all places, in every season, and all sorts of 'constitutions' of the year; in the open country, as well as in the confined city. It does not appear to seize more readily any particular constitution or habit of body. The robust as well as the slender and weak,—the delicately secluded female, who goes not abroad but in a glazed carriage, and steps only on woollen carpets,—and the sturdy country boy, trudging a mile to school, through rain and mud, or amidst ice and snow,—all appear equally liable to this distemper. Nay, farther, children belonging to the same household, suffering at the same time under the same disorder, though differently circumstanced, come out in the end pretty much alike." (p. 67.)

In this chapter we are first let into another of our author's peculiar views concerning whooping-cough,—viz. that it is an inflammation *sui generis;* a sort of *exanthema* of the mucous membrane of the œsophagus and cardiac portion of the stomach.

The next chapter, entitled "*of the Breathing and Vocal Organs*," contains some most extraordinary doctrines, clothed in some of the most transatlantic English we ever remember to have seen. We must give our readers some specimens, premising that the aim of the whole chapter is to show "that cough is no more a proof of disease existing in the lungs, than shedding tears can be supposed to indicate that the cause of distress is in the eyes: in other words, that the derangement, or "*labefaction*," of distant parts, is often expressed through the vocal organs; though he admits that these organs, by being very often and for a long time called on to express the pain and morbid affections of that complicated machine, the human body, may at length become actually disordered and half worn out.

The windpipe, says Dr. Waterhouse, (page 76,) is the speaking trumpet of our internal nature. A man, under the punishment of the lash, expresses his agony through the natural vocal organ.

"In the ticklish, irritable, and glowing hectical state, the same thing happens, but with more intensity. Sudden noise, quick surprise, a thought, a recollection, an inconscious association of ideas,—nay, the scratching off the head of a pimple, a dream, may excite a cough, that shall be for a few minutes irresistible. Sometimes we see the *hectic of genius* in both sexes,—that rapid action which accompanies precocity of intellect, where there is neither lesion nor labefaction of any organ—producing similar effects.

"Whenever the pituitary membrane of the nostrils is vellicated to sneezing, the tubes, ducts, and vesicles of the respiratory organs are consentaneously affected; and it is as natural for the membranes lining the breathing and vocal organs to cough, as for the membrane of the nostrils to sneeze: one is no more a mark of disease than the other (p. 77.)

This is certainly a *fine* specimen of *fine* writing, but it sinks into insignificance before the following:

"The larynx is covered with a mucous membrane, which, at its entrance, is endowed with extreme sensibility. But it is almost impossible to have an accurate idea of the larynx, unless we could see its operation in the living human subject. The nerves, vessels, cartilages, slender muscles, and ligaments of the vocal organs, are so numerous and complicated, and their nomenclature so overloaded with verbosity, that they serve to confound the understanding, and overwhelm the pupil in undiscerning wonder, rather than to fill his mind with just conceptions of the parts and their uses. *Surprise and humiliation must be the portion of the anatomist, when he considers that every individual has a tone of voice peculiar to himself.*" (p. 79.)

So far from this, we should think the anatomist would be a great deal more surprised if people were all to talk in the same tone. What there is to *humiliate* him, either in the one or in the other, is to us quite incomprehensible.

The last extract we shall venture upon from this curious chapter is remarkable for the manner in which the concluding thought is expressed, hardly inferior to the specimen last before us. Speaking of a notion of HOFFMAN's, that cough sometimes arose from an inverted flow of humours, Dr. Waterhouse remarks, that this skilful author believed "that whooping-cough arose from causes which produce in some habits rheumatism, and not from a specific contagion. It is remarkable that the sagacious Sydenham entertained a similar opinion respecting dysentery. It is before such authority that ordinary physicians hesitate. Most of us are destined to follow rules; others, rules must follow them." (p. 80.)

Our author, in his sixth chapter, enters upon the consideration of the appearances in diseased bodies after life: and here, by the way, we could not help remarking that Dr. Waterhouse, displeased probably with that awkward *hybrid* term, now so common with all our writers, (and not a few of our reviewers,) —we mean "*post-mortem appearances*,"—has tried to mend it by adopting "*post vitam appearances.*" We really doubt which is the most inelegant of the two.

Upon the whole, this chapter has satisfied us better than any other in the book. It contains a number of very shrewd and useful observations on morbid appearances, and the degree of importance to be attached to the finding of them or otherwise. The author has evidently here studied in a good school, and we quote with pleasure the following reflections:

"Should we pass from the centre of the body to the head, we shall find that appearances after life frequently turn out very different from the predictions before it departed. Sometimes we find a congestion of blood where we expected water; and sometimes the vessels of the pia mater distended, appearing like a beautiful injection; and see in the brain itself, when divided by a knife, a number of red points exuding blood; and yet no inflammation was expected there. At other times we discover the arachnoid web separated from the pia mater, by the intervention of water; and sometimes these membranes lose their transparency, without any very remarkable derangement of the intellect; while the principal weight of alteration of the organic structure was found to be in the stomach and bowels." (p. 96, 97.)

"If death occasion such an alteration in the visible parts, how much greater must be the alteration in the invisible and most subtle organization of our bodies, to the integrity of which life adheres? I hope not to be misunderstood: I would by no means discard the evidence of morbid anatomy. I would only request the practitioner to weigh what has been said, before he pronounces confidently on the causes of death in the body after life is departed. The most acute and experienced anatomists, the most sagacious physiologist, skilful surgeon, and profound physicians, feel the greatest diffidence in these all important subjects. They bear in mind the subtilty of nature, and the imperfections of the senses. When discussing these matters, they should not forget that they are sitting in judgment on something which has the softness of air in receiving impressions, and the activity of fire in exerting its actions." (p. 99.)

Dr. Waterhouse brings forward cases to show that whooping-cough may occasion death in a variety of ways; sometimes by an affection of the lungs, (pages 94 and 95,) and sometimes by abdominal disorganizations, (page 91.) He might have added also by affections of the brain.

The seventh chapter treats of the operation of *remedies*, deducing thence the nature of whooping-cough; and it opens by the query—"Have not all those medicines, most efficacious in mitigating the severity of whooping-cough, a special reference to the alimentary canal, rather than to the trachea, bronchiæ, or *(pneumatical)* system of the lungs?" (p. 101.)

Our readers doubtless know enough of medical treatises not to require to be told that, however they may differ in matters of theory, they generally agree wonderfully in their practical suggestions. Yet it may perhaps surprise them, remembering our author's melancholy reflections on the "*instability of European practice* in this disease," to find him thus expressing himself:

"Select emetics, judiciously administered, are of prime importance in this disorder." (p. 101.) "All our best writers recommend purgatives in whooping-cough." (p. 102.) "There can be no doubt of the frequent, though short-lived, efficacy of blisters; and their operation tends to strengthen our opinion that the proximate or exciting cause of this tussis convulsiva is an exanthematous affection of certain membranes, extra pulmonic." (p. 109.) "There can be little doubt of the efficacy of cantharides and balsam of capivi in relieving, and sometimes curing, whooping-cough." (p. 109.) Embrocations with tartar emetic are spoken of favourably in page 110. In the next page we learn that "millipedes have been given with good effect, and that too many in this day believe them good for nothing." "Prussic acid appeared beneficial in several cases; it merits further trial." (p. 111.) "Equal portions of linseed oil and flower of sulphur has been used with remarkable success in mitigating the violence of whooping-cough. Some people consider it the sovereign remedy in this distressing complaint. A tea-spoonful of this mixture, taken three or four times a-day, or less frequently, according to the age of the subject, has certainly produced beneficial effect." (p. 112.)

Now we really should like to know what Dr. Waterhouse can find to pride himself upon in all this? It appears to us much the same sort of opinion as has been given in all ages, and by all authors who have ever written on whooping-cough. We merely set out by a different road; and our readers are now prepared to say whether they prefer it to the spasmodic views of Cullen, the bronchial theory of Dr. Watt, or the cerebral notions of Dr. Webster? For our own parts, we do not pretend to understand accurately what Dr. Waterhouse would wish us to believe. The basis of his superstructure is very extensive, and, if one part of it fails, he may still cling to the remainder. The assigning to whooping-cough a seat so extensive as the præcordia, appears to us very little better than calling it at once a disease of the *general system*, which brings us back very nearly to the opinions of Willis, and not far from those of Cullen. But to couple this with the notion of an exanthematic eruption about those parts appears to us to be little better than theory run mad. We have no direct proof of such a thing taking place, (as the author himself allows;) and his analogical proofs, drawn from the efficacy of blisters, are too trifling to merit discussion.

Upon the whole, we rose from the perusal of the work sadly disappointed. All that we remained satisfied of was, that there still exists something about whooping-cough which is very difficult of explanation; but this we well knew before, and, instead of having our minds cleared concerning the nature of these difficulties, we continued, at the close of our labours, rather more cloudy on the subject than when we sat down. A sort of confused jumble between stomach and brain, œsophagus and bronchia, lungs and bowels, respiration and digestion, spasm and inflammation, flitted before our fancies; and it was long before we could dissipate the sort of *fog* in which this oddly-compounded book had enveloped our understandings.

There still remain three chapters unnoticed. The one entitled "Analogy between Whooping-cough and Dysentery;" the next "on the Management of Children;" and the last treats of the power of vaccination in removing or mitigating whooping-cough. We noticed nothing in either of these chapters particularly deserving praise or censure.

It remains only that we say a few words concerning the author's style. A few American idioms may have been already observed; as, for instance, "the membrane of the lungs being *vellicated to sneezing*," (p. 77.) A few other instances of the same kind, however, occurred to us while engaged in reading the work; and we select the following for the edification of the curious:—In p. 63, we read "our knowledge of nature is too limited for *speculative positivity*." Again, p. 67, "Children *cosseted* in the nursery pass through the disease much in the same way as others who reject aid and refuse medicine;" or, lastly, (p. 84,) "The dentition appeared to *progress* with the reluctance of a backward vegetation."

We wish the author would settle in his own mind whether he will make *lungs* take rank in the singular or plural number. The following sentence is a sad proof of the want of some such understanding.

> "In this species of chronical affection, the lungs expresses *its* sympathy, by now and then a deep inspiration, or sigh, and now and then by a rending cough. The causes continuing from season to season, the lungs at length partake so much of the primary affection of the stomach, intestines, liver, and probably spleen, that *they*, on great exposures, or some serious cause of mental distress, sink into a state that ends like all other consumptions." (p. 82.)

But it is painful to go on finding faults. We do not deny that there are some good reflections scattered through the work, but we think (and, from the specimens which we have adduced, our readers will probably agree with us,) that there is nothing in it calculated to raise, in any considerable degree, the fame of American pathologists.

---

Art. III.—*Memoria sull' Idrocele del Cordone Spermatico.* Di Antonio Scarpa, Professore Emerito et Direttore della Facoltá Medica, nell I. R. Universitá di Pavia, &c. &c.

*Memoir on the Hydrocele of the Spermatic Cord.* By Antonio Scarpa, Honorary Professor and Director of the Faculty of Medicine in the University of Pavia, &c. &c. &c. With two Plates. 1823.

The illustrious Professor Scarpa, in the present Memoir, has undertaken to clear up this point of practical surgery, by the assistance of pathological anatomy, the true and only method of extending the boundaries of our art. With this intent, he has annexed to his work two copper-plates, executed in a masterly manner, and which demonstrate plainly the different forms of the hydrocele of the spermatic cord, conducting us to a secure diagnosis of the malady, and suggesting the most convenient method of cure; on which accounts, we consider it necessary to present the student of surgery with a short account of this memoir, which, by explaining the doctrines and recording the experience of the author in these complaints, will serve to rectify the errors, and to correct the vague notions, which have too much prevailed relative to their real nature and seat.

Two of the most ancient Greek physicians, LEONIDAS and PAULUS EGINETA, were acquainted with that form of watery tumor of the scrotum, known by the name of hydrocele of the cord; distinguished its double form; and were aware of the difference between it and the hydrocele of the vaginal coat of the testicle,—a distinction which, although of the greatest importance, had been overlooked by CELSUS, GALEN, and ORIBASIUS. FALLOPIUS revived the doctrine of Paul of Egina, which had been neglected by Albucasis, as well as by the other Arabian interpreters and commentators on the Grecian writers. In modern times, the progress of anatomy, the frequency of post-mortem examinations, and the accurate observations made at the patient's bedside, have thrown a stronger light upon this subject, especially as far as regards the structure and connexion of the parts containing and contained within the scrotum, and consequently upon the exact seat of each of these diseases called *watery herniæ.* Thus, it is known now that the tunica rubicunda and vaginalis do not form one membrane, but are distinct, the first being called the cremaster; the dartos is no longer ranked as one of the proper tunics of the testicle; it is now known that no cavity exists between the dartos and the cremaster, nor between the lamina of the vaginal coat, nor between the tunica albuginea and the thin pellicle which covers it. In former days, the reciprocity of the vaginal coat with the parts placed within and without the great sac of the peritoneum before and after birth, and in the different states and changes of life, were unknown; from which sources of information are derived the more exact diagnosis of the maladies of the scrotum and testicle in general, and particularly of the watery collections that take place within its cavity; and, in the second place, the useful modifications made by modern surgeons in the ancient methods of cure formerly practised for the radical cures of these complaints.

In these researches, WISEMAN, HUNTER, BERTRANDI, DOUGLAS, and POTT, have particularly distinguished themselves: the last, indeed, describes the two distinct forms assumed by the hydrocele of the spermatic cord so exactly, that in that respect he has left us scarcely any thing to desire. Nevertheless, it cannot be dissembled that, whether it be owing to the infrequency of the disease, or the difficulty of applying precepts to practice, the well-instructed surgeon often feels perplexed and at a loss to decide upon the real nature of the complaint, especially in those cases where the hydrocele of the cord is complicated with that of the tunica vaginalis on the same side of the scrotum, or where one of the forms of the dropsy of the cord constitutes with the testicle a mass nearly uniform, and from whence a difficulty arises of distinguishing one from the other.

In his memoir upon *Hernia*, our author has taught that the cellular membrane placed behind the great sac of the peritoneum surrounds the spermatic vessels, passes out with them through the inguinal ring, and accompanies them even to their insertion into the testicle; besides which, that, from the groin to the bottom of the scrotum, the spermatic vessels, with their cellular envelope, together with the vaginal coat, are surrounded and enclosed within the musculo-aponeurotic sheath of the cremaster. Thus, upon the occasion of the throwing out of serum in the cellular texture which invests and accompanies the spermatic cord, the water, passing from cell to cell, occupies and swells up the spermatic cord, sometimes their whole length from the loins to the scrotum, sometimes only from the groin to their insertion into the testicle. This swelling, more or less extended, on account of the manner in which the water is diffused, is called the *diffused hydrocele* of the spermatic cord.

In dissecting this first species of hydrocele of the cord, the parts present themselves in the following order:—The integuments of the scrotum being laid open vertically down to the dartos, the musculo-aponeurotic sheath of the cremaster first presents itself, more or less thickened and compact, according as the disease is more or less voluminous or inveterate. Immediately under this is found the dense cellular involucrum of the cord, tumid with water, which is contained both within and on the outside of it, and which at first sight does not look unlike the hernial sac formed by the peritoneum. Pursuing the dissection still farther into the heart of the tumor, from its intimate texture, much serosity escapes, as it were from a sponge; and in consequence of which the tumor lessens little by little, and finally disappears altogether, either spontaneously or with the assistance of slight pressure. All the water being discharged, the blood-vessels of the spermatic cord are distinctly seen, which were at first confounded in the mass. The cells of the above-mentioned spongy substance, which in the healthy condition of the cord are scarcely visible to the naked eye, are, in the case of aqueous effusion, converted into a mass of little bladders filled with water, some of them sufficiently large to admit the point of the finger. This vesicular structure is not found throughout the whole extent of the cord, in very large or inveterate cases of the disease, because, in proportion as the tumor descends towards the bottom of the scrotum, the cells burst and disappear altogether, especially in the lowest part of the swelling, where there is found nothing but a simple cavity filled with water; and it is on that account that, in the large diffused hydrocele of the cord, the fluctuation is only clearly discernible towards the base of the swelling; and it is only in that direction that a certain quantity of water can be extracted in a continued stream, by means of a puncture,—a circumstance, however, not common to small and recent cases of this description. The serum of this form of the hydrocele is generally limpid and thin, sometimes yellowish, greenish, or albuminous; in some rare instances it was found to be gelatinous, so that the whole cellular substance surrounding the spermatic vessels appeared converted into gelatine, which gave way under the fingers.

However large and inveterate this form of the disease may be, the base is always limited to the point of the insertion of the spermatic vessels in the testicle, or, at the most, a very little below it; and this is the reason why the diffused hydrocele of the cord never induces any remarkable displacements of that gland from its accustomed situation. On this account the surgeon, both by the sight and touch, will always discover the testicle in its place: nay, the base of the tumor is always distinguished from the testicle by a manifest semilunar groove, variable, however, as to its depth and width in the same subject, according to the greater or less contraction of the cremaster. These circumstances are distinctly shown in the plates.

If, in the body of a subject affected with the diffused hydrocele of the cord, the tunica vaginalis be opened on the same side, and a finger introduced on the internal and lower side of that tunic, a strong septum will be found, which hinders all communication between the base of the hydrocele and the cavity of the tunica vaginalis. On this pathological fact is founded the just and secure diagnosis of this complaint, and from which are formed the principal signs by which it is to be distinguished from the hydrocele of the vaginal coat.

As soon as the above-mentioned disease has acquired a considerable size, although it does not occasion great pain, it becomes an object of uneasiness to the patient, and of attentive consideration for the surgeon. At first the tumor is of a cylindrical form, which afterwards becomes pyramidal; but, however large it may become, it never causes the penis to appear so much retracted under the pubes, as in the case of a hydrocele of the tunica vaginalis of equal size. The diffused hydrocele of the cord is little, if at all, sensible to pressure, but yields under the finger as a vesicular body full of water would do, not without some degree of elasticity. If there be fluctuation, this is not evident, excepting at the lower part: there, if the fluid be compressed by the hand, it ascends towards the groin, but slowly, and not without effort: on the contrary, under the same degree of pressure, the liquid in the vaginal coat readily mounts to the top of the tumor, and distends it. In the diffused hydrocele, moreover, the testicle is seen, and may be felt below the base of the tumor; which never can be done with the eye, nor ever by examining the base of the hydrocele of the tunica vaginalis with the hand.

When the diffused hydrocele of the cord occupies and distends the inguinal ring, it is not easy to distinguish it from an inguinal omental hernia, although Pott was of a different opinion. The difficulty, however, remains now as great as formerly, because both tumors occupy the same situation; they are both cylindrical at first, and afterwards assume a pyramidal form; both are soft and flexible; neither of them possess much sensibility; and they are both reducible, with some difficulty, within the cavity of the abdomen. The perplexity is, however, greatly increased if such a case as is mentioned by Pipelet should occur, in which the portion of omentum protruded had been converted into hydatids, or into a mass of small bladders filled with water, as is the case in the incipient stage of the diffused hydrocele of the cord. Neither is the non-return of the omental hernia, when replaced, (the patient being in a horizontal position,) always a certain and infallible diagnostic mark, because the author has found the contrary to take place in some cases of small omental herniæ, either because it had only been forced within the inguinal canal, or else drawn down by some portion of the same viscus, which had formed an adhesion to the hernial sac. M. Scarpa thinks it proper to mention this difficulty, and to confess at once that, upon this point, our art is defective; and the surgeon, therefore, can only supply this defect by circumspection in pronouncing the diagnosis, and caution in performing an operation.

The diffused hydrocele of the cord is sometimes associated with that of the tunica vaginalis of the same side of the scrotum, a circumstance observed both by Leonidas and Paulus. In this complication, the tumor has an appearance more irregular than when the disease is simple. The extraordinary volume of the neck of the tumor, with the remarkable dilatation of the ring, are especially to be observed. Of the two distinct hydroceles of the same side, that of the vaginal coat is anterior, and descends lower down; the other is posterior, and is a little inclined towards the external margin of the scrotum. The anterior is also distinguished from the posterior by the interposition of a furrow, which runs obliquely upon the anterior part of the scrotum at different distances from the bottom of it, according to the greater or less distention of the vaginal coat; that is to say, towards the lower part of the scrotum, where the quantity of water is but small, and towards its summit, where it is considerable.

The above-mentioned signs are sufficient to make known this case of hydrocele; but, should any shadow of doubt remain, the surgeon may clear it up by puncturing the anterior tumor, since the water which distended the tunica vaginalis being removed, if a diffused hydrocele of the cord exists at the same time, that will remain full and distended as before, and discover itself immediately.

In his memoir upon Hernia, our author has described a particular congenital formation of the tunica vaginalis, in which, when distended with water, it assumes the shape of two tumors placed one upon the other, on the anterior face of the scrotum, divided nearly in the middle by a circular mark. This is, however, an illusory appearance, and they are really but one, which is easily known, because the groove is in the middle, and not at the top; and also because, if the lower tumor is pressed, the fluid flows into the upper one, and, if the inferior one be punctured, both are emptied, the water flowing in a continued stream. Morgagni is inclined to think that the water effused and retained in the cellular texture which surrounds the spermatic cord may, by its weight and acrimony, break down the division between the base of the hydrocele and the cavity of the tunica vaginalis, and thus form one instead of two hydroceles of the same side. Garengeot and De la Faye were of the same opinion; but our author does not believe that the annals of surgery afford one clear example of this disease, in the sense entertained by the above-named writers. But the possibility of a communication being formed between the two hydroceles, in consequence of ulceration of the testicle extending to the boundary dividing them, is amply proved by the result of the history given by Munro, and referred to in our author's work. But the water, whether collected by effusion or defective absorption, contained in the cellular texture round the spermatic cord, does not always spread equally from cell to cell, so as to occupy the whole extent of the cord, or that which reaches from the ring to the body of the testicle. It happens sometimes that the serosity stops within a limited number of cells,—sometimes immediately above the testicle, sometimes in the middle of the scrotum, sometimes a little beneath the abdominal ring; and appears enclosed, as it were, in a little compact membranous bag, like an encysted tumor; from whence this species of hydrocele of the cord has obtained the name of the *encysted hydrocele*.

Le Dran and Bertrandi have seen these watery cysts, or tumors, disposed along the cellular envelope of the spermatic cord, like so many follicles or hydatids. The encysted hydrocele has also been observed in females, in the cellular membrane which surrounds the round ligament of the womb, and accompanies it out at the ring into the groin and pudendum, and which is sometimes called, though improperly, the *canal of Nuch*. Dessault and Lallemand had occasion to see this species of hydrocele, which is also described by Paré; but, what is still more extraordinary, the midwife Aspasia, in the most remote age, gives an accurate description, and which will be found referred to in the works of Ætius Tetra, B. iv. cap. 100.

The encysted hydrocele of the spermatic cord, which appears above the testicle, is commonly of an oval shape. As its volume is usually small, it at first appears to form only one substance with the testicle; but, examined attentively, it is observed to be distinct from it, unless, indeed, the testicle be morbidly enlarged, softened, or disorganised. But the diagnosis is not so easy when this tumor is of a large size, and situated in the same place, because part of the testicle is, as it were, imbedded in the watery tumor, but not so as to escape the tact of an experienced surgeon: on which account, if that portion of the tumor which projects forwards from the bottom of the scrotum, and a little laterally, is found to be of a moderate consistence, flexible, smooth, and, above all, sensible even to slight pressure, whilst the rest of the tumor has only the marks and character of a bladder full of water, it may be safely asserted that the first lower portion of the swelling is part of the sound testicle, and the remainder is the encysted hydrocele of the spermatic cord. The whole mass of the tumor is distinguished from schirrus, or cancer of the testicle; since it is not hard, nor uneven and knotty, nor subject to occasional darting pains.

The cyst of the above-described watery tumor is composed of two strata; that is, of the musculo-aponeurotic sheath of the cremaster, and, beneath, of the cellular membrane of the spermatic cord, more or less thickened. The external surface of this capsule is overspread with arterial and venous branches; the internal surface is irregular, fringed, or like velvet. The fluid contained within is most commonly limpid, albuminous, sometimes yellow or greenish, and rarely of a reddish cast, like the leys of wine; a circumstance, from whence has arisen the mistake of those who have taken the disease for Melicera. The pressure made by the tumor on the testicle merely forces it a little lower in the scrotum, and a little forward: nevertheless, the author found, in the dead body of a man who had this disease, the testicle in a state of atrophy, and the tunica vaginalis adherent to it; but without being able to discover which disease had the priority in point of time,—that of the testicle or the encysted hydrocele of the cord. This disease sometimes commences at an intermediate distance above the testicle. In its commencement, the tumor has only the appearance of a small varicose knot in the cord itself; but finally it increases, and obtains the size of a pigeon's egg. Uninformed surgeons are too apt to mistake this for a third testicle; and it is wonderful, after what LEONEDES, PAUL EGINETA, and FALLOPIUS, have written upon this point, that such men as GRAAF, BARTHOLIN, FERNELIUS, FORESTUS, ROLFINCIUS, &c. have fallen into the same error.

The encysted hydrocele of the cord, whatever distance it may be placed above the testicle, is moveable in all directions, as if it were attached to a pedicle; and, pushed upwards, it raises and carries up with it the testicle. This little tumor, in its origin, occasions some uneasiness, but afterwards causes no sort of trouble: it gives no pain, unless pressed firmly with the finger, together with the spermatic vessels. The distance of the tumor from the testicle varies every instant, from the irregular contractions of the cremaster, and the variable corrugations of the skin: in other respects, it is flexible, elastic, and, being touched, gives the sensation of containing a fluid, as before mentioned.

In this place our author records some observations, which prove the pathological facts above mentioned, and confirm the practical inferences deduced from them; and, from the first observation, it follows, that, when the diffused hydrocele of the cord is met with, together with that of the tunica vaginalis of the same side, although the former is but in an incipient state, the best method is to open them both at the same time; since experience proves, that sooner or later the operation will be necessary, since neither of them are capable of being terminated in any other way. Besides, this operation is never attended by serious consequences, at least when the disease is purely local. But, unfortunately, the diffused hydrocele is occasionally accompanied with unfavourable circumstances, both local and general; for example, when, besides its being of long standing, it extends from the bottom of the scrotum to the loins, or attacks subjects advanced in life, or affected with any general or visceral disease. In these cases, simple as the operation is, it exposes the patient to great danger, and even risks the loss of life. The same observation also applies to some cases of hydrocele of the tunica vaginalis; a truth which Dodoneus, Fallopius, and Frank, have established.

But, besides these general causes, which prevent the happy termination of this operation in either case of hydrocele, a disordered condition of the intestinal canal is, according to our author, the most formidable of the whole. WISEMAN, who first insisted upon this, cautions surgeons, that the radical cure of hydrocele, in the phthisical, in the scrofulous, in those affected with œdema of the feet, with yellow complexions, and cachetic habits, is followed by great pain in the abdomen, by fevers, vomitings, hiccough, and gangrene; and which remark was confirmed both by CHESELDEN and SHARP. This doctrine has been further extended by BERTRANDI; and to these reasons POTT added the diseased condition of the aponeurosis of the cremaster and the tunica vaginalis, as causes of the disasters that sometimes followed the operation; but, according to our author, this effect is rather to be attributed to the general sensibility of the testicle itself in certain cases, when exposed to the action of external agents, since he has never observed this effect to follow when the tunica vaginalis is opened to remove coagulated blood, because it is then covered and defended by a stratum of blood, which the prudent surgeon will not remove. LATTA says, that, to remove all risk, the best way would be, after having evacuated the water, to put the tunica vaginalis into close contact with the tunica abuginea, and to maintain them in that situation by the assistance of art. This, says our author, is true; but it must be recollected that the tunica vaginalis, although healthy, is not always susceptible of union in every point of its surface, and frequently that is the case in consequence of tubercles, fungosities, or hydatids; and he therefore recommends the testicle to be covered with fine linen overspread with wax and oil, and to leave this covering until it is thrown off by suppuration, leaving the albuginea covered with granulations, and rendered less sensible to the contact of air and external agents. This is the practice which, in these cases, has always answered the expectations of the celebrated Professor SCARPA.

---

ART. IV.—*Leçons sur les Epidemies et l'Hygiene publique.* Par F. E. FODERÉ. Tom. II.—pp. 565. Paris, 1823.

THE second volume of M. FODERÉ's work has been published much sooner than we anticipated, and it will be speedily followed by a third, which completes his design. In a short address to his readers, our author takes occasion to give a sly hit at the physiologists, *par excellence*, the counter-stimulants, as well as those who have experimented upon living animals, and upon the nature of poisons; and he concludes, that the study of sound medicine consists solely in the observation of natural phenomena. In conformity with this belief, he not only set about restudying the works of HIPPOCRATES himself, but actually caused his son to translate that great man's work upon Epidemics word for word, in order that he might compare his own descriptions with those of the Father of Medicine; and he felicitates himself upon finding the most perfect accordance between them. The only drawback to his satisfaction appears to be, that a necessary increase of expence is thus incurred to the editor, (how, we do not exactly perceive,) and which the subscribers, he trusts, will not fail to take kindly.

The volume contains the remaining diseases of the first order,—namely, those produced by food or drink; the whole of the second order, comprised in three chapters, on those epidemics caused by miasma, or other effluvia; and the third order, consisting of six chapters, containing the diseases produced by variations in the temperature of the air, or from the dry or moist condition of the atmosphere.

The first chapter in the volume is the third of the first order, and is devoted to a consideration of *Raphania*, or fevers with convulsion, and *Ergotism*, or that disease caused by the use of the ergot, or damaged rye. This chapter contains much information upon the subject of which it treats; but it is not one of great interest to the English reader. Rye forms but a small part of the national food; and we certainly do not meet with epidemic diseases that are at all traceable to the impurities of our bread-corn, though some adulterations must be admitted, we fear, to be pretty general. The term Raphania, it appears, was first bestowed by LINNÆUS upon fevers of a spasmodic character, in consequence of his belief that they originated in the use of the grains of the *raphanus raphanistrium*, and always placed these fevers in the typhoid class. Though the name of raphania is still continued, the same kind of affection is said equally to be produced by the mixture of the *broma multiflora*, the *darnel*, and other plants, with the *cerealia*, as well as by the use of damaged corn.

M. Foderé cites many examples of epidemics caused by the use of the ergot of rye, especially in France; some of them, indeed, of a very recent date.

Passing this chapter, we come next to the consideration of *epidemic diarrhœa*, of which M. Foderé only acknowledges the following species,—viz. the saburral, the worm, the bilious, the serous, and the mucous diarrhœas; and which may be considered, we think, amply sufficient, when it is considered that our author is treating of epidemic diarrhœas only, and not those which are simply the signs or the symptoms of other complaints. We do not, however, think it necessary to dwell at any length upon the symptoms, causes, or modes of cure, of this simple complaint, because M. Foderé distinctly confines his remarks in this chapter to the mere frequency of dejections, and observes that, when the system suffers and fever is present, or where the stomach rejects the food and bilious vomitings occur, the complaint is not diarrhœa, but cholera morbus in the one case, or dysentery in the other. Among the causes, the sudden change of atmosphere at certain seasons of the year, (and which, in this country, we must acknowledge to be the principal source of the summer, or rather autumnal, diarrhœa, so prevalent at that season;) but this chapter most decidedly shows the defect of our author's arrangement, since the disease he treats of is classed among those produced by the effects of food or drink; and yet we find checked transpiration of the skin as one of the most frequent and prominent causes of the complaint.

The fifth chapter, on *dysentery* and *dysenteric fever*, is a very favourable specimen of M. Foderé's style, and contains many important remarks, the result evidently of much personal observation; and the practice recommended is, in our opinion, generally speaking, highly rational and judicious. The extent of the volume does not permit us to quote largely; but we shall translate a passage or two from this chapter, to confirm the judgment we have ventured to pronounce: but we must, *in limine*, object, as usual, to our author's division and subdivision of this malady, since the mere *intensity* of a disease is not a reasonable ground for giving it an additional name; and there does not appear to us to be any good reason for saying more than that dysentery is either sporadic or epidemic, and that it is sometimes complicated with fevers of the typhoid character, and at other times not so.

The following is M. Foderé's description of the disease in its most common form:—Occasional chills, or a general sense of coldness, which continues for some hours; paleness of the face; a weight at the stomach; lassitude; nausea; sometimes vertigo; slight colicky pains about the navel, followed by liquid and yellowish evacuations, which are at first abundant; at the termination of one or two days, more or less, these stools no longer occur, but the pains in the abdomen increase in frequency and intensity. At length, with tenesmus, and with a marked expression of countenance, which has been denominated a pinched countenance, *(face gripée,)* a small evacuation is voided, which affords no relief, and which is composed of a glairy matter mixed with blood. The colour of these dejections varies much; they are either brown, green, black, &c. more or less liquid, and of a peculiar fœtor. The pains preceding each evacuation augment, and then become more frequent, even to the amount of eight, ten, twelve, or fifteen, in the hour. The stools occasionally contain worms, at other times substances resembling portions of the intestines, and sometimes clots of blood. The tenesmus is frequently followed, especially in infants, by a prolapsus ani, which must always be carefully and immediately replaced. The violence of the pain will sometimes produce palsy of a limb, as in the painter's colic. The urine is scanty in quantity, and strangury often occurs. This disease frequently remits for some hours in the day, only to become aggravated in the evening, and especially at night; and it has been said to be always of a febrile nature. This rule is, however, too general. It must be admitted that there is almost always an augmentation of heat, a dryness of the skin and tongue, thirst, and some frequency of the pulse, which is small and contracted; but it is also true that many patients, labouring under this disease, are evidently without fever, and are even less altered than by a common diarrhœa. (p. 79.)

This last sentence, by the bye, is a direct contradiction to what our author has previously said in his chapter on diarrhœa.

M. Fodere enters next into a consideration of the causes of dysentery, and afterwards, speaking of the contagious nature of the disease, is of opinion that it is contagious under certain circumstances; and he supports his opinion by some strong facts, and particularly from what he witnessed at the Hospital of Entrevaux, where the old patients in the hospital were attacked with the dysentery which had become epidemic among the troops cantoned in the neighbourhood.

Our author's practice appears to be judicious, and we are glad to find that he acknowledges the advantages of general blood-letting in the first attack, if the person be robust, or of local bleeding in those of more debilitated habits; a practice, the benefit of which we have had many occasions of witnessing in the course of the last war. Indeed, with the exception of, perhaps, something like prejudice against purging, our author's mode of treatment is deserving of high commendation; and he truly says, there is no universal anti-dysenteric remedy, but the treatment must vary according to its degree, as well as complication, (p. 101;) and we only regret that our narrow limits oblige us to curtail our extracts, and to pass on to the sixth chapter, devoted to the consideration of *scurvy and scorbutic affections;* a wide field, and one which recent circumstances in this country have rendered doubly interesting to us.

Commencing with a pretty long definition of this malady, and one which leads us to believe that it is still of very common occurrence in France, our author proceeds to say that scurvy may be considered as the first degree of the decomposition of organisation during life, characterised by lassitude, heaviness, alteration of the complexion, an itching, redness, pain and spongy swelling of the gums, with a disposition to bleed, and looseness of the teeth; a fetid breath, stiffness of the hams, swelling of the legs, with purple or lead-coloured spots on the extremities or other parts of the body; commonly without fever, and without any sensible loss of the intellectual faculties. M. Foderé condemns all subdivisions of this malady, and says that it is always the same, only differing according to the temperament and strength of the subjects attacked by it, or by its complication with other diseases. (p. 118.)

Our author distinguishes four periods of the disease, and gives it as his opinion that the palagra of Lombardy is nothing more than a particular species of scurvy; in which opinion he is supported by the authority of many Italian physicians.

Without stopping to examine into the question of the contagious nature of this malady, we will next point out the result of the post-mortem examinations of those who have fallen victims to its ravages. The most marked appearances are the rapid decomposition of the body; the blood presents no coagulum, and is capable of being taken from the body by the opening of one vessel only; the muscles softened and flabby; the bones softened, and separated from their cartilages; the lungs gorged with blood; the heart flaccid, and greatly dilated; a good deal of serosity in the pericardium and the thoracic cavities; the peritoneum, and its duplicatures, frequently covered with black spots; the mucous membrane of the intestinal canal affected in the same way; the mesenteric glands tumefied, obstructed, and sometimes in a state of suppuration; the brain, and its appendages, always sound. M. Foderé paid particular attention to the state of the blood of those affected with scurvy during life. That drawn from the arm at the termination of the first period, instead of separating into two distinct parts, it displayed a singular mixture of dark-coloured and red stripes. He also examined the infectious matter produced on the gums of these patients, and he found it composed of animal mucus, impregnated with a fetid odour, of an ammoniacal muriate, of sulphuretted lime, and an acid, which he believed to be the hydrocyanic. Ammonia especially, the ordinary product of putrefaction, was found in great quantity, (p. 131.)

A few words will suffice to explain the causes of scurvy. Bad nourishment, excessive fatigue, very cold and moist air, or warm and moist air, ennui, fear, and in general all the depressing passions united in the same subject, or only in part affecting them. We cannot afford room to treat of these causes in succession; but they are worthy of perusal, and are certainly the result of ample experience on the part of the author.

Passing then, unwillingly, over many pages of valuable matter, we find our author insisting (p. 151,) upon the impossibility of overcoming this disease by the powers of nature alone: but it would be no less an error, he continues, to suppose that, like syphilis, it is always curable by one specific remedy; for example, by antiscorbutic vegetables. On the contrary, rich broths, and the flesh of the turtle, have often been of the greatest use, between the tropics, in these affections; and in the scurvy at Paris, no surer method of overcoming the malady was found, than that of administering *good wine, and doubling the rations.* These words of wisdom might have been applied, perhaps beneficially, at no distant period, in our own country.

We cannot venture to give any further extracts from this chapter; but we may remark, that M. Foderé's curative means are chiefly directed to diet, and to a change of air, where it can be procured. Under certain circumstances, and with proper limitations, he does not altogether proscribe blood-letting in the first period of this complaint; but he thinks, and we apprehend with reason, that it ought not lightly to be resorted to. He condemns the common prejudice of the utility of tobacco as a preservative; and he does not say one word as to the utility of mercury as a remedy in this disease. The chapter terminates with some handsome compliments to Captains Parry and Ross, and to Captain Phillibert, whose respective voyages were performed with as few sick, or perhaps fewer, than would be found to prevail among the same number of people in the ordinary occupations of life.

We have now arrived at the *second order* of diseases, namely, those arising from miasma or other effluvia; the first chapter of which, comprising fifty-two pages, is devoted to simple intermittent fever produced by miasma, or independent of it; marked fevers; and periodic painful affections. *(neuroses periodiques.)* M. Foderé treats first of regular *intermittent fever*, observing upon the almost infinite variety of their types; which scholastic division, he remarks, is of little importance, the nature of the malady remaining the same in all of them. After describing the usual mode of attack, and the common routine of symptoms in these diseases, our author goes on to remark the enlargement of the spleen, which appears to belong exclusively to this class of maladies, and which, in the fever of Walcheren, we have had occasion to witness in numerous instances: on one occasion the spleen, when taken out of the body, weighed five pounds and a quarter avoirdupois.

In treating of the causes of intermittents, M. Foderé places the effluvia of marshes very justly in the first rank; but he contends that exposure to wet and cold, sleeping in wet clothes, &c. will, under certain circumstances, produce this disease; a point which we will readily concede. Neither shall we dispute the justice of his enumeration of the occasional causes; but we scarcely know how to agree with him in thinking that the proximate cause of intermittent fever consists in "a sub-irritation of the spinal marrow, from whence the whole nervous system is sympathetically affected," (p. 193;) and which is proved, he thinks, by the shiverings which arise in this part of the body, and by the spasms and other accidents which accompany the cold stage of the fever. What the French call marked fevers are, in fact, intermittents, which in the first instance assume the character of affection of some particular organ, such as the hemicrania, periodical tooth-aches, colics, &c. &c.; and the true nature of which is only ascertained by the regular return of the symptoms at determinate periods.

Passing over a long discussion with regard to the corruptive or depuratory nature of these fevers, a question much agitated formerly, and still continuing to divide the opinions of continental practitioners; we have no hesitation in asserting, with M. Foderé, that, with very few exceptions, all fevers are *corruptive*, and that the duty of the physician is to bring them to a termination as speedily as possible. We need not detail the means of cure laid down by our author: with the exception of an over-confidence in the virtues of the *divine* quinquina, as he terms it, we have found nothing especially to attract our notice.

M. Foderé informs us, that his principal object in writing the second chapter on *fievres subintrantes*, and those of an insidious character, was to keep the attention of the practitioner fixed upon those septic principles which are either continually emanating from situations acknowledged to be unwholesome, or which are merely the result of those necessary combinations which the elements of all organic and inorganic bodies, either upon or under the earth, are subject to. These principles which, he says, have vaguely and dangerously been designated as special epidemic constitutions, depending upon something unknown, some *quid divinum* in the air, are not only capable of producing particular diseases, but also of altering the nature of those which have arisen from other causes.

The greater part of this chapter is devoted to an explanation of terms, and which we in this country do not usually employ, such as *fievres trompeuses*, *insidieux*, *pernicieuses:* terms which are so vague, and so little explanatory of any of the pathognomonic signs of fever, that we are surprised to find them dwelt upon and adopted by our author. He makes a distinction also between remittent fevers and those which he denominates *subintrantes*, which, to inattentive observers, he says, present the aspect of continued fevers; whereas, they are composed of repeated accessions of fever, a fresh one coming on before the preceding one has entirely subsided. We shall not fatigue our readers with pursuing this explanation further; nor can we quite agree with M. Foderé, that the year 1649, the period when the Peruvian bark was first introduced into Europe, has been equally fortunate for humanity with the year 1799, when vaccination was first regularly announced as a preservative against small-pox. In this chapter, indeed, as in most of those composing the volume, the *methodus medendi* appears to us infinitely less valuable than the accurate descriptions of disease, and the many valuable practical remarks connected therewith.

The third chapter treats of *remittent fevers*, and especially those of warm climates. These fevers, says M. Foderé, are observed to assume different forms: sometimes there will be two remissions in twenty-four hours, at others only one ; sometimes there is a remission on the first and third days only. In this chapter little is said as to treatment: bleeding, a purgative, and the bark, appear to be the *ne plus ultra* of our author's practice; but there are some good precautionary rules laid down for those who are obliged to visit and reside in those unwholesome climates where remittent fevers are found annually to prevail.

[To be continued.]

